# Evaluating Frontline Health Workers’ Responses to Patient Inquiries With and Without Large Language Model Support in Nigeria: Observational Study

**DOI:** 10.2196/86578

**Published:** 2026-11-05

**Authors:** Maria Moosa Munnee, Oluwaseyi Malumi, Solomon Chinedu, Wilson Ike-Odoe Okah, Ayoposi Ogboye, Chiagozie Abiakam, Thameshree Duys, Khushi Jain, Imo Etuk, L Nneka Mobisson

**Affiliations:** 1 mDoc Healthcare, 1A Hakeem Dickson Drive Off T.F. Kuboye Street, Lekki Phase 1, Lagos, Nigeria, 234 8103160497; 2 Independent Researcher, Lagos, Lagos, Nigeria; 3 Department of Systems Engineering, Faculty of Engineering, University of Lagos, Lagos, Lagos, Nigeria; 4 Department of Research, British Institute in Eastern Africa, Nairobi, Kenya

**Keywords:** ChatGPT, large language models, low- and middle-income countries, LMIC, AI, frontline health workers, community health workers, physicians, patient safety, Nigeria

## Abstract

**Background:**

Frontline health workers in low- and middle-income countries (LMICs) often face barriers that compromise care quality, including limited training, high patient loads, inadequate access to updated clinical guidance, and resource constraints. Working in underserved settings further exacerbates challenges to providing accurate, complete, and empathetic responses. Addressing these gaps requires innovative tools to support FLWs in real time.

**Objective:**

This study aims to evaluate the effectiveness of ChatGPT-3.5 (OpenAI) in enhancing the quality of FLWs’ responses to health inquiries in Lagos, Nigeria.

**Methods:**

In this observational study, 36 licensed frontline health workers (doctors, nurses, and community health workers) practicing general or primary care in Lagos, Nigeria, generated responses to 15 patient-generated health questions covering maternal and neonatal health, family planning, and sexually transmitted infections. Each FLW produced responses using their own knowledge and available resources (human-only) and with ChatGPT-3.5 assistance (GPT-aided). A panel of 4 clinicians evaluated responses using a 5-point Likert scale for accuracy, empathy, contextualization, completeness, safety, and overall preference. In addition, 8 nonclinician health seekers assessed a subset of responses across 3 themes: Caring, Trust, and Ease of Understanding.

**Results:**

Across 1080 responses from 36 frontline health workers, GPT-aided responses significantly outperformed human-only responses on all clinician-rated metrics (*P*<.001), with the largest gains observed in completeness, empathy, and overall preference, particularly among community health extension workers (CHEWs). Nonclinician evaluators also favored GPT-aided responses across all themes, with a strong preference for trust.

**Conclusions:**

This study provides empirical evidence that a large language model (ChatGPT-3.5) enhances the completeness, empathy, and perceived trust of responses by frontline health workers in Lagos, Nigeria. These findings underscore the potential of integrating ChatGPT-3.5 to strengthen health care delivery in resource-limited settings, highlighting the necessity for capacity building and further investigation into its safe, equitable, and effective implementation.

## Introduction

AI is increasingly being explored as a tool to help health care workers provide services to their patients, particularly in regions where poor health systems, health care workforce shortages, and infrastructure limitations pose significant challenges, such as sub-Saharan Africa [1]. Several studies have objectively compared the quality of care provision at health facilities across different African countries. These studies consistently highlight persistent challenges such as inadequate infrastructure, workforce shortages, and inconsistent service delivery that undermine the quality of care [2-4]. Additionally, people often express dissatisfaction with the quality of care at health facilities; factors such as prolonged wait times and lack of equipment contribute to their dissatisfaction and the subpar quality of care they receive [5]. Systematic reviews from Kenya and other countries highlight deficits in clinical care, patient safety, and outcomes, underscoring the urgent need for innovative approaches to strengthen service delivery [3,4]. These findings provide important context for exploring how emerging technologies like AI can support health care workers in improving service delivery, particularly in resource-limited settings.

In Nigeria, structural barriers including economic instability, limited infrastructure, and a shortage of skilled health care workers (1.83 per 1000 people, far below the World Health Organization [WHO]–recommended 4.45) contribute to delayed access to preventive care and high rates of avoidable morbidity [6-9]. Amidst these challenges, AI, specifically large language models (LLMs), shows promise in digital health’s role in potentially lessening systemic barriers [10]. LLMs are being deployed for various uses in health care delivery, along with their own risks and rewards [11]. Due to the vast amount of data upon which they are trained (and later, fine-tuned for specific applications), these models lead in their pattern discernment, predictive capacity, and ability to understand context as they successfully carry out tasks and prompts [12]. LLMs have aided clinical workflow through text and concept extraction, summarization and synthesis, automating communication, drafting relevant treatment plans, as a supplementary educational resource, and much more [13]. Recent studies in low-resource African settings have shown the potential of LLMs to support frontline health care delivery, ease workflow demand, optimize clinical performance, and enhance care quality [14,15]. Nonetheless, several concerns regarding hallucinations, bias, and lack of specialized skills to use them highlight the necessity of rigorous evaluation to safeguard patient safety [16].

ChatGPT (OpenAI), one of the most widely used LLMs with over 300 million weekly active users as of January 2025 [17,18], generates near-human-quality text but was not designed for health care [1,19]. Its use is growing among clinicians and health care professionals in low- and middle-income countries (LMICs) such as Nigeria, often without clear information about its risks, benefits, or limitations [20]. While discourse often focuses on AI replacing health care workers, emerging research suggests AI may be better suited to complement and support them [21,22,23]. Despite this growing interest and adoption, empirical evidence on the safe integration of LLMs into frontline health workers’ workflow is scarce.

To evaluate the potential of LLMs as a complementary tool in frontline workflows, this study compared the effectiveness of frontline health workers in Lagos, Nigeria, when responding to women’s health-related questions, covering maternal and neonatal health, family planning, and sexually transmitted infections (STIs), using standard clinical resources vs ChatGPT-3.5. The study is part of a broader mDoc Health care initiative to integrate LLMs into women-centered care. We hypothesize that responses generated with ChatGPT support would perform better in terms of accuracy, empathy, safety, contextualization, completeness, and overall preference than responses produced using standard clinical resources alone. Findings from this work provide early insights into how LLMs might be safely and effectively incorporated into clinical workflows in low-resource settings.

## Methods

### Study Design

This observational study, conducted in March 2024, evaluated how the use of ChatGPT-3.5, the free, publicly available version last trained in 2022, influenced frontline health workers’ responses to common primary care questions from health seekers in Lagos, Nigeria. Responses generated with ChatGPT-3.5 assistance (GPT-aided) were compared with those produced using standard clinical resources (human-only) across 6 domains: Accuracy, Empathy, Contextualization, Completeness, Safety, and Overall Preference. Standard clinical resources refer to any materials they commonly use in routine clinical practice, including clinical guidelines, textbooks, or internet-based resources, excluding LLMs. The study also examined both clinicians’ and nonclinicians’ preferences for GPT-aided vs traditional responses. Participants accessed ChatGPT-3.5 through their own devices and OpenAI accounts. Throughout this study, “GPT” refers specifically to this version, and “participants” refers to the 3 frontline health worker groups, including medical doctors (MDs), registered nurses (RNs), and community health extension workers (CHEWs). The overall study design and workflow is highlighted in Figure 1.

**Figure 1.**
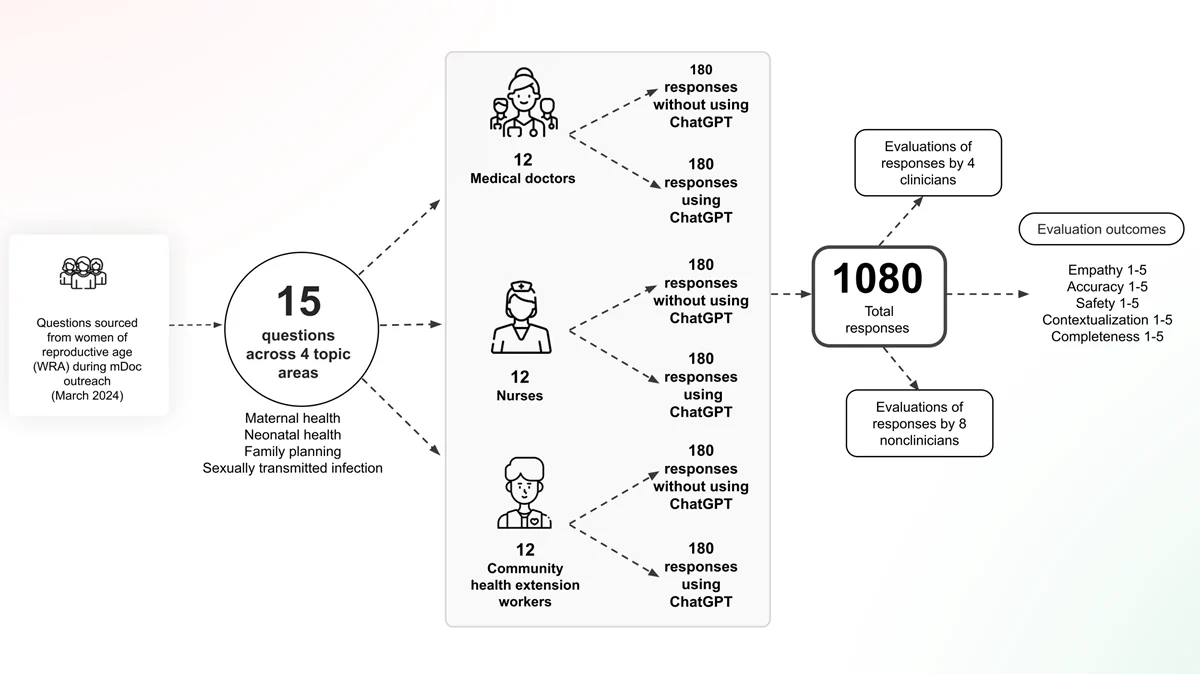
Study design and workflow for generating and evaluating frontline health worker responses to patient health inquiries with and without ChatGPT (Open AI) support in an observational study in Lagos, Nigeria (2024).

### Setting and Participants

The study enrolled 36 licensed frontline health workers in Lagos, Nigeria: 12 MDs, 12 RNs, and 12 CHEWs. All participants were actively practicing general medicine or primary care in public or private facilities. Eligibility required professional licensure: MDs (MBBS or MBChB degrees and registration with the Medical and Dental Council of Nigeria), RNs (Bachelor of Nursing Science [B.N.Sc] or Diploma or Ordinary National Diploma and RN certificate from the Nursing and Midwifery Council of Nigeria), and CHEWs (National Diploma from School of Health Technology, Community Health Practitioners Registration Board of Nigeria) [24]. Frontline health workers in specialized roles were excluded. Recruitment was conducted via open calls, and the study was implemented in person in Lagos.

### Question Selection

In March 2024, 15 frequently asked questions (Multimedia Appendix 1) were drawn from conversations between mDoc health coaches and women of reproductive age during an outreach event in Lagos. mDoc is a digital health social enterprise based in Lagos, Nigeria, that provides end-to-end, AI-enabled self-care health coaching to members living with or at risk of chronic conditions, as well as individuals with general health and wellness needs. mDoc has supported over 180,000 members, the majority of whom are low-income and more than 80% of whom are women. These questions spanned 4 health topic areas (maternal health, neonatal health, family planning, and STIs). They reflected authentic, recurring health concerns raised during ongoing coaching interactions, ensuring that the study addressed issues directly relevant to women’s lived experiences. The restriction to these health topics is in line with the health focus of the larger study within which this study is nested, and reflective of the commonly asked health queries by mDoc’s members.

### Measurements and Sample Size Justification

Each frontline health worker answered the 15 questions twice: once using their standard resources (human-only) and once with ChatGPT-3.5 assistance (GPT-aided), yielding 30 responses per participant and 1080 responses overall (36 frontline health workers×30 responses). Participants were provided with 2 Google Forms with instructions and questions added. They first completed the human-only responses using their own expertise and any resources they typically consult in clinical practice, excluding the use of any LLMs. After completing this task, participants answered the same questions with assistance from ChatGPT-3.5. Participants were not provided with a standardized prompt template or formal training. Instead, they were instructed to enter the patient’s question into ChatGPT and use the tool in a manner they deemed appropriate to generate their response. This approach was chosen to observe how frontline health workers would naturally interact with an LLM in a high-pressure, real-world environment without specialized training.

They were asked to upload screenshots of their conversations with ChatGPT. Most participants entered the questions into ChatGPT verbatim, while some rephrased them based on their interpretation of the query. In both conditions, participants were instructed to ensure responses were accurate, empathetic, safe, complete, and contextually relevant to Nigeria.

The target sample size of 1080 responses was determined based on a priori power calculations to detect a 10 percentage point difference in key response metrics (Accuracy, Empathy, Safety, Contextualization and Completeness, and Overall Preference). For the power analysis, the Likert scale ratings of the 6 response metrics were conservatively dichotomized into binary outcomes, classifying responses with scores ≥4 considered as meeting the quality threshold and scores ≤3 classified as below the threshold, assuming a baseline proportion of 50% for either human-only or GPT-assisted responses [25,26]. Using a 2-sided alpha of 0.05 and 80% power, approximately 776 responses (388 per condition) were estimated to be required under the assumption of independent observations to detect the specified difference; our sample exceeded the target. However, this calculation assumed independence across all responses and did not account for the intraclass correlation arising from the 30 responses contributed by each of the 36 participants. Accordingly, the target sample size of 1080 responses should be interpreted as a minimum feasibility estimate rather than a formally cluster-adjusted sample size.

Participants completed a survey administered in 2 stages. Prior to the response-generation tasks, a presurvey captured demographic characteristics, clinical experience, use of digital communication channels, and prior exposure to LLMs, using a combination of categorical and Likert-scale items with response-specific anchors. After completing both the human-only and GPT-aided response tasks, participants completed a postsurvey assessing their experience with ChatGPT, including perceived ease of use, usefulness, efficiency, and trust compared with traditional resources. Postsurvey responses were measured using 5-point Likert-scale items with response anchors specific to each construct (Table 1).

**Table 1.** Self-reported experiences and perceptions of the use of ChatGPT (Open AI) among community health extension workers (CHEWs), medical doctors, and registered nurses in an observational study in Lagos, Nigeria (2024).^a^

	CHEWs^b^ (n=12)	MDs^c^ (n=12)	RNs^d^ (n=12)	Total (n=36)	*P* value
Have you previously ever used a large language model?, n (%)					.10
I don’t know	1 (8.3)	0 (0.0)	2 (16.7)	3 (8.3)	^e^—
No	6 (50.0)	1 (8.3)	5 (41.7)	12 (33.3)	—
Yes	5 (41.7)	11 (91.7)	5 (41.7)	21 (58.3)	—
Which large language models have you used?, n (%)					—
ChatGPT	5 (41.7)	11 (91.7)	5 (41.7)	21 (58.3)	—
If you have ever used large language models (eg, ChatGPT) have you ever used them for medical or clinical purposes?, n (%)					.03
I have never used large language models	7 (58.3)	1 (8.3)	7 (58.3)	15 (41.7)	—
ChatGPT but not for medical purposes	4 (33.3)	6 (50.0)	1 (8.3)	11 (30.6)	—
ChatGPT for medical purposes	1 (8.3)	5 (41.7)	4 (33.3)	10 (27.8)	—
Have you ever conducted formal telemedicine consultations?, n (%)					.22
No	6 (50.0)	2 (16.7)	4 (33.3)	12 (33.3)	—
Yes	6 (50.0)	10 (83.3)	8 (66.7)	24 (66.7)	—
On average, how much time does it take you to answer a patient’s question?, n (%)					.57
0‐5 minutes	6 (50.0)	7 (58.3)	5 (41.7)	18 (50.0)	—
6‐10 minutes	2 (16.7)	3 (25.0)	6 (50.0)	11 (30.6)	—
11‐15 minutes	1 (8.3)	1 (8.3)	0 (0.0)	2 (5.6)	—
16‐20 minutes	2 (16.7)	0 (0.0)	1 (8.3)	3 (8.3)	—
More than 20 minutes	1 (8.3)	1 (8.3)	0 (0.0)	2 (5.6)	—
Do you answer health questions for health seekers/patients through digital channels (eg, answering questions via SMS or WhatsApp or Telegram or Email, etc)?, median (IQR)	5.00 (2.75-5.00)	3.50 (3.00-5.00)	4.00 (3.75-5.00)	4.00 (3.00-5.00)	.59
How important do you feel it is for licensed health care professionals to refer to evidence often when answering patient questions?, median (IQR)	5.00 (5.00-5.00)	5.00 (5.00-5.00)	5.00 (3.75-5)	5.00 (4.75-5.00)	.30
On average, how often do you refer to external resources before answering patient/health seekers’ questions via digital channels?, median (IQR)	3.00 (2.75-3.5)	3.00 (2.75-3.25)	3.50 (3.00-4.00)	3.00 (3.00-4.00)	.40
Please rate your level of skill in obtaining or gathering clinical information, median (IQR)	5.00 (4.00-5.00)	4.00 (4.00-5.00)	4.00 (4.00-4.00)	4.00 (4.00-5.00)	.20
Based on your experience, how common were the questions asked in the survey?, median (IQR)	5.00 (4.00-5.00)	4.00 (4.00-5.00)	4.00 (3.00-5.00)	4.50 (4.00-5.00)	.23
Based on your experience, how difficult were the questions in the survey?, median (IQR)	5.00 (4.00-5.00)	4.00 (3.00-4.00)	4.00 (3.00-4.00)	4.00 (4.00-5.00)	.007
ChatGPT was easy to navigate, median (IQR)	5.00 (5.00-5.00)	5.00 (4.75-5.00)	5.00 (4.00-5.00)	5.00 (4.75-5.00)	.63
When using ChatGPT, it would be easy to get confused, median (IQR)	1.00 (1.00-1.00)	1.00 (1.00-1.25)	1.50 (1.00-2.00)	1.00 (1.00-2.00)	.19
ChatGPT understood my inputs well, median (IQR)	5.00 (4.00-5.00)	5.00 (4.00-5.00)	5.00 (4.00-5.00)	5.00 (4.00-5.00)	.96
ChatGPT failed to recognize a lot of my inputs and prompts, median (IQR)	1.00 (1.00-2.00)	1.00 (1.00-2.00)	1.50 (1.00-2.25)	1.00 (1.00-2.00)	.51
ChatGPT’s responses were useful, median (IQR)	5.00 (5.00-5.00)	5.00 (4.00-5.00)	5.00 (4.00-5.00)	5.00 (4.00-5.00)	.53
ChatGPT’s responses were irrelevant, median (IQR)	1.00 (1-1.25)	1.50 (1.00-2.00)	1.00 (1-1.25)	1.00 (1.00-2.00)	.53
ChatGPT coped well with any errors or mistakes, median (IQR)	4.00 (3.00-5.00)	4.00 (3.75-4.25)	4.00 (4.00-5.00)	4.00 (3.00-5.00)	.83
ChatGPT’s responses helped make my responses more accurate to the best of my knowledge, median (IQR)	5.00 (4.00-5.00)	4.00 (3.00-5.00)	4.00 (4.00-5.00)	4.00 (4.00-5.00)	.24
ChatGPT’s responses helped make my responses more complete to the best of my knowledge, median (IQR)	5.00 (3.75-5.00)	4.00 (3.00-5.00)	4.00 (4.00-5.00)	4.00 (4.00-5.00)	.43
ChatGPT helped me be faster in generating my response, median (IQR)	5.00 (3.75-5.00)	4.00 (4.00-5.00)	5.00 (4.00-5.00)	5.00 (4.00-5.00)	.67
ChatGPT had little effect on my response, median (IQR)	2.00 (1.00-4.25)	2.00 (2.00-3.00)	1.50 (1.00-3.00)	2.00 (1.00-3.00)	.60
I sometimes disagreed with some or all of ChatGPT’s responses, median (IQR)	1.00 (1.00-1.25)	2.50 (2.00-3.25)	2.50 (1.00-3.00)	2.00 (1.00-3.00)	.01
I am willing to use the responses provided by ChatGPT, median (IQR)	5.00 (4.00-5.00)	4.00 (4.00-5.00)	5.00 (4.00-5.00)	4.50 (4.00-5.00)	.36
I am more comfortable using my usual resources instead of ChatGPT, median (IQR)	2.00 (1.00-2.25)	3.00 (2.00-3.00)	2.00 (1.00-3.00)	2.00 (1.00-3.00)	.35
I trust my usual resources more than ChatGPT for clinical work, median (IQR)	2.00 (1.00-3.00)	3.00 (3.00-4.00)	2.00 (1.00-3.00)	3.00 (2.00-3.00)	.009

a Items were scored on 5-point Likert-type scales using construct-specific response anchors: digital channel frequency: 1=never, 5=all the time. Importance of evidence: 1=not important, 5=very important. External resource referral frequency: 1=never, 5=every time. Skill in gathering clinical information: 1=very poor level of skill, 5=very skilled. Question commonality: 1=very uncommon, 5=very common. Question difficulty: 1=very easy, 5=very difficult. ChatGPT usability and perception items: 1=strongly disagree, 5=strongly agree.

b CHEW: community health extension worker.

c MD: medical doctor.

d RN: registered nurse.

e Not applicable.

### Evaluation and Outcomes

To assess the quality and perceived value of frontline health workers’ responses, with and without ChatGPT assistance, a dual-panel evaluation was conducted using clinical experts and nonclinicians (Table 2).

**Table 2.** Demographic characteristics of clinical (n=4) and nonclinical (n=8) evaluators in an observational study assessing frontline health worker responses with and without ChatGPT support in Lagos, Nigeria (2024).

Demographics	Clinical evaluators (n=4)	Nonclinician evaluators (n=8)
Sex, n (%)		
Female	3 (75)	8 (100)
Male	1 (25)	—
Age (years), n (%)		
18‐24	—	1 (12.5)
25‐34	—	5 (62.5)
35‐44	2 (50)	2 (25)
45‐54	1 (25)	—
55 years and above	1 (25)	—
Highest academic degree, n (%)		
Bachelor’s (MBBS, B.N.Sc, etc)	4 (100)	6 (75)
No degree	—	2 (25)
Specialty, n (%)		
Pediatrics	1 (25)	—
Nursing	1 (25)	—
Family medicine	1 (25)	—
Obstetrics and gynecology	1 (25)	—
Years of experience, mean (SD)	18 (9.07)	—

1. Clinical panel: This panel consisted of 4 licensed experts (3 physicians and 1 nurse) who assessed the responses via a structured rubric covering 6 domains: Accuracy, Empathy, Safety, Contextualization, Completeness, and Overall Preference (Multimedia Appendix 2). All but Accuracy and Overall Preference included a “Not Applicable” option. Ratings used a 5-point Likert scale with written justifications. Before evaluation, evaluators attended a virtual orientation to standardize application of the rubric. Responses were anonymized, randomized, and blinded by response type. Evaluators had 5 days to complete scoring. They were encouraged to work in multiple shorter sessions and take breaks as needed.
2. Nonclinical panel: To capture user-centered insights, 8 nonclinicians from mDoc’s member base were randomly selected from individuals who expressed interest in participating after being notified of the opportunity by their health coaches and who met the inclusion criteria, including being women of reproductive age, fluent in English, and having at least a secondary school education. The panel was fully blinded to the source of the health responses, seeing only option “A” or “B” without knowledge of whether an answer was generated by an MD, RN, CHEW, or ChatGPT-3.5.

Due to the high volume of the primary data pool (N=1080), a subset of 256 responses (128 GPT-aided and 128 human-only) was selected for nonclinician preference evaluation via stratified random sampling. To ensure thematic breadth, 2 questions were selected from each of the 4 clinical domains: maternal health, neonatal health, STIs, and family planning. For each selected question, responses from 16 frontline health workers were sampled in a 6:6:4 ratio (6 MDs, 6 RNs, and 4 CHEWs). This structured approach ensured that the nonclinician preference data accounted for variability in both clinical content and the baseline expertise of the respondents across all professional groups. Evaluation followed a paired-comparison format, where each of the 128 response pairs was independently evaluated by all 8 nonclinician evaluators, resulting in a total of 1024 individual evaluation points.

For each pair, evaluators selected their preferred response (A or B) based on 3 specific themes:

1. Caring: “Which response do you feel was more caring?”
2. Trust: “Which response do you trust more?”
3. Ease of Understanding: “Which response do you feel is easier to understand?”

This aimed to reveal broad preference trends across FLW types (MDs, RNs, and CHEWs), without testing for small effects.

### Statistical Analysis

To objectively assess the structural and linguistic characteristics of the responses, we performed a series of computational text analyses. Lexical volume was measured via word counts, and text readability was quantified using the Flesch-Kincaid Grade Level metric (via the Python textstat library, version 0.7.4). Additionally, sentiment analysis was performed using the TextBlob natural language processing library (version 0.19.0) to determine the polarity (ranging from negative to positive) and subjectivity of the responses.

Demographic characteristics of the frontline health workers were summarized using frequencies and percentages. For bivariate analyses, categorical variables (eg, age range and gender) were compared across the 3 frontline health worker groups (MDs, RNs, and CHEWs) using *χ*² tests, with Fisher exact tests used where expected cell frequencies were small (n<5).

#### Clinician Evaluation

Clinical evaluation scores (Accuracy, Empathy, Safety, Contextualization, Completeness, and Overall Preference) were summarized using medians and IQRs, as Shapiro-Wilk tests and visual inspection histograms confirmed a nonnormal data distribution. Because each of the 36 frontline health workers generated both human-only and GPT-aided responses for the same set of questions, and multiple independent clinical raters evaluated these outputs, the study design yielded repeated measurements clustered within respondents and evaluated by multiple raters.

To account for the repeated-measures structure of the data (responses generated by 36 unique frontline health workers and evaluated by 4 independent raters), inferential analyses were conducted using linear mixed-effects models (LMM). Condition (GPT-aided vs human-only) was included as the primary fixed effect. Frontline health worker respondent was specified as a random effect (36 clusters), and rater was included as a fixed-effect covariate given the small number of rater levels (k=4). Models were fitted using maximum likelihood estimation. Subgroup analyses were conducted by stratifying models by professional license cohort. Overall distribution of clinician evaluators’ ratings across response type and evaluation metrics was visualized using violin plots. To assess the degree of agreement among multiple independent clinical raters evaluating response quality, Fleiss’ Kappa statistics were computed for each evaluation metric.

#### Nonclinician Evaluation

Nonclinician preference data were analyzed as binary outcomes derived from paired comparisons between GPT-aided and human-only responses. For each evaluation theme (Caring, Trust, and Ease of Understanding), selections favoring GPT-aided responses (B) were coded as 1 and those favoring non-GPT responses (A) as 0.

Descriptive preference percentages were calculated as the proportion of selections favoring GPT-aided responses out of the total number of individual evaluator assessments (N=1024, representing 128 pairs evaluated by 8 evaluators). Preference proportions were summarized as overall percentages and stratified by frontline health workers professional group who authored the response (MDs, RNs, and CHEWs). To account for the clustered structure of the data, inferential analyses were conducted using generalized estimating equations (GEE) logistic regression with evaluator (Rater ID) specified as the clustering variable and an exchangeable working correlation structure. Results are reported as odds ratios (OR), 95% cCIs, and 2-sided Wald *P*-values. Statistical significance was defined as *P*<.05.

#### Survey Analysis

Categorical variables were summarized using counts and percentages. Ordinal Likert-scale responses were reported as medians and IQR, using 5-point response-specific anchors tailored to each construct (Table 1). Bivariate comparisons across the 3 FLW groups (MDs, RNs, and CHEWs) were conducted using *χ*² or Fisher exact tests (where n<5) for categorical variables, and the Kruskal-Wallis test for Likert-scale items.

All statistical analyses were conducted in Python (version 3.13; Python Software Foundation), and Stata (version 18; StataCorp LLC), with significance levels set at *P*<.05. Data was visualized to illustrate the distribution of responses across professional categories.

### Ethical Considerations

Ethical approval was obtained from the Lagos State Health Research Ethics Committee (LSHREC/2023/0011) and the Federal Capital Territory Health Research Ethics Committee (FHREC/2023/01/140/01-08-23). The study was also conducted in accordance with the guidelines established in the approval protocol for human participant involvement. Written informed consent was obtained from all individuals who took part in the study before commencement. The participants were informed of the purpose and objectives of the study, as well as their right to voluntary participation, which could be withdrawn at any point during the study. To protect the privacy and confidentiality of the participants, no personally identifiable data was used (materials or images), and all data collected were deidentified before being used for analysis. All data is securely stored, with restricted access only to authorized team members. Additionally, to cover transport expenses, participants received a modest stipend, which varied by participant depending on the traveling distance, ranging from NGN 20,000 to NGN 50,000 (US $1=NGN 1327.38, as of October 2026).

## Results

### Overview

The majority of participants were female (25/36, 69.4%), with a statistically significant difference in gender distribution across frontline health worker groups (*P*=.03) (Table 3). Age distribution also varied significantly (*P*=.03), with the majority of participants across all 3 groups falling within the 25‐34 age range. Notably, no MDs fell within the 35‐44 age range, compared to 33.3% (4/12) of CHEWs and 16.7% (2/12) of RNs, reflecting a younger age profile among medical doctors in this sample. Educational attainment differed significantly (*P*=.003), with 41.7% (5/12) of RNs holding bachelor’s degrees compared to 25.0% (3/12) of CHEWs. Years of experience likewise varied across groups (*P*=.01, Kruskal-Wallis test), with CHEWs having the highest median of 4.00 (IQR 4.00-6.75) years, followed by RNs (median 3.00, IQR 1.85-4.00) and MDs (median 2.00, IQR 2.00-2.70). Most participants (30/36, 83.3%) had between 1‐5 years of experience, with only 6 exceeding 5 years. Overall, two-thirds of participants (24/36, 66.7%) reported having conducted formal telemedicine consultations. Additionally, half of the respondents (18/36, 50%) stated that they typically answer patient queries in under 5 minutes (Table 1).

**Table 3.** Demographic characteristics of community health extension workers (CHEWs), medical doctors, and registered nurses who generated responses to patient health inquiries with and without ChatGPT (OpenAI) support in an observational study in Lagos, Nigeria (2024).

	CHEWs^a^ (n=12)	MDs^b^ (n=12)	RNs^c^ (n=12)	Total (n=36)	*P* value
Age (Years), n (%)					.03
18‐24	0 (0.0)	1 (8.3)	4 (33.3)	5 (13.9)	—
25‐34	8 (66.7)	11 (91.7)	6 (50.0)	25 (69.4)	—
35‐44	4 (33.3)	0 (0.0)	2 (16.7)	6 (16.7)	—
Sex, n (%)					.03
Female	9 (75.0)	5 (41.7)	11 (91.7)	25 (69.4)	—
Male	3 (25.0)	7 (58.3)	1 (8.3)	11 (30.6)	—
Highest academic degree, n (%)					.003
Diploma	7 (58.3)	0 (0.0)	4 (33.3)	11 (30.6)	—
Ordinary national diploma	2 (16.7)	0 (0.0)	3 (25.0)	5 (13.9)	—
Bachelor’s (MBBS, B.N.Sc, etc)	3 (25.0)	12 (100.0)	5 (41.7)	20 (55.6)	—
Years of practice, n (%)					.06
1‐5	8 (66.7)	12 (100.0)	10 (83.3)	30 (83.3)	—
6‐10	2 (16.7)	0 (0.0)	0 (0.0)	2 (5.6)	—
11‐15	2 (16.7)	0 (0.0)	2 (16.7)	4 (11.1)	—
Years of practice, median (IQR)	4.00 (4.00-6.75)	2.00(2.00-2.70)	3.00 (1.85-4.00)	3.00 (2.00-4.25)	.01

a CHEW: community health extension workers

b MD: medical doctor.

c RN: registered nurse.

Survey results indicated diverse insights into participant experiences and perceptions with ChatGPT (Table 1). Most rated themselves as skilled or very skilled in gathering clinical information, and 58.3% (21/36) had prior LLM experience, mainly with ChatGPT (21/21, 100%). However, only 27.8% (10/36) had used it for clinical tasks. All agreed on the importance of evidence-based responses. They found ChatGPT user-friendly, easy to navigate, and understood inputs well, and that it enhanced response speed, accuracy, and completeness. Still, MDs and RNs were more likely to disagree with ChatGPT responses compared to CHEWs (*P*=.01) and trusted their usual clinical resources more (*P*=.009). While most frontline health workers were open to using ChatGPT, the findings suggest participants balanced the tool’s benefits with caution and reliance on established practices, reflecting measured enthusiasm for integrating AI into their workflows.

### Clinician Evaluator Scores Analysis

The score distribution for GPT-aided (green) and human-only (orange) responses across 6 evaluation metrics is illustrated in Figure 2. For Accuracy and Safety, both GPT-aided and human-only were rated highly, with scores clustering around 4 and 5; however, GPT-aided responses show a slightly wider distribution toward the upper end, suggesting greater preference. Empathy scores were widely spread for both conditions, with GPT-aided responses being slightly more concentrated at a higher rate. For Contextualization and Completeness, GPT-aided and human-only responses scored similarly around 4, though GPT-aided responses display a wider spread towards 5, suggesting a slight preference.

**Figure 2.**
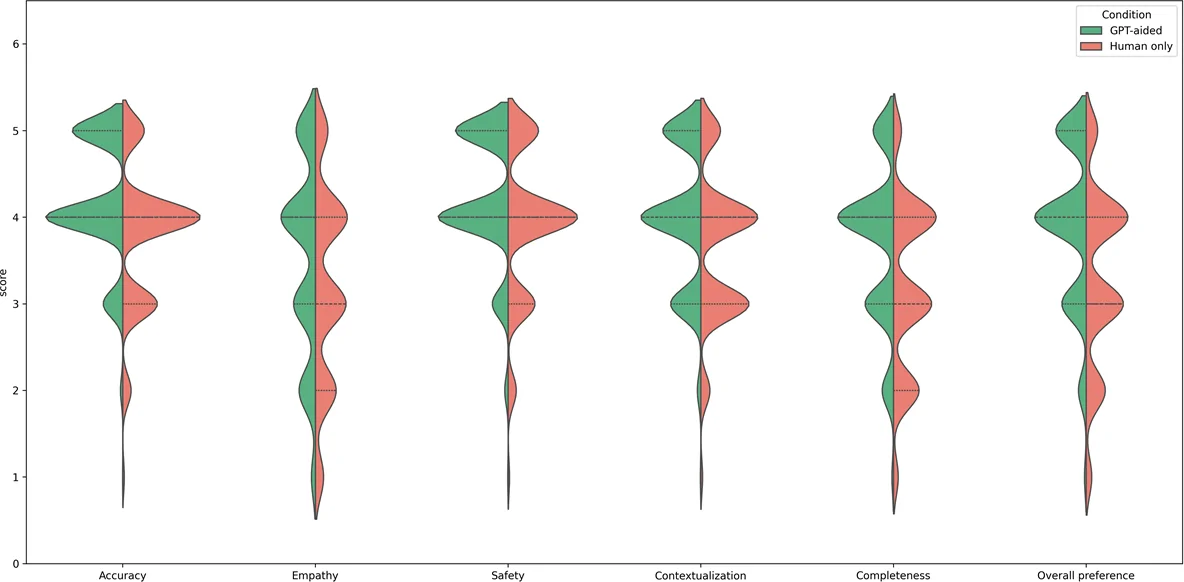
Distribution of clinician-evaluated scores for GPT-aided (green) vs human-only (orange) responses across 6 quality metrics, generated by 36 frontline health workers in an observational study conducted in Lagos, Nigeria (2024). Violin plots display the distribution and variability of Likert-scale ratings assigned by clinical evaluators to paired responses.

Consistent with these distributions, median scores were equal or higher for GPT-aided responses across all metrics (Table 4). Median Accuracy and Safety scores were identical between conditions (median 4.00, IQR 4.00-5.00 for GPT-aided vs median 4.00, IQR 3.00-4.00 for human-only responses), although the GPT-aided IQRs were shifted toward the higher end of the rating scale. GPT-aided responses received significantly higher ratings (*P*<.001) for Empathy (median 4.00, IQR 3.00-4.00 vs median 3.00, IQR 2.00-4.00), Completeness (median 4.00, IQR 3.00-4.00 vs median 3.00, IQR 2.00-4.00), and Overall Preference (median 4.00, IQR 3.00-5.00 vs median 3.00, IQR 3.00-4.00) compared to human-only responses. Human-only responses showed wider variability for Empathy and Completeness, whereas GPT-aided responses showed greater variability for Overall Preference. Contextualization scores were identical in median (4.00, IQR 3.00-5.00 for GPT-aided vs 4.00, IQR 3.00-4.00 for human-only responses) but showed greater variability for GPT-aided responses. Linear mixed-effects models indicated significantly higher clinician ratings for GPT-aided responses across all 6 evaluation domains (all *P*<.001).

**Table 4.** Comparison of clinician-evaluated GPT-aided and human-only responses across 6 evaluation metrics reported as median (IQR) from paired responses generated by 36 frontline health workers in an observational study in Lagos, Nigeria (2024).^a^

Metric	GPT-aided, median (IQR)	Human-only, median (IQR)	*P* value
Accuracy	4.00 (4.00-5.00)	4.00 (3.00-4.00)	<.001
Empathy	4.00 (3.00-4.00)	3.00 (2.00-4.00)	<.001
Safety	4.00 (4.00-5.00)	4.00 (3.00-4.00)	<.001
Contextualization	4.00 (3.00-5.00)	4.00 (3.00-4.00)	<.001
Completeness	4.00 (3.00-4.00)	3.00 (2.00-4.00)	<.001
Overall preference	4.00 (3.00-5.00)	3.00 (3.00-4.00)	<.001

a Descriptive statistics are presented as median (IQR). *P* values were obtained from linear mixed-effects models comparing GPT-aided and human-only responses, with frontline health worker respondent included as a random effect and clinical rater included as a fixed-effect covariate.

Differences across frontline health worker groups (CHEWs, RNs, and MDs) examined in Table 5 highlight variation in the magnitude of improvement with GPT support. Accuracy and Safety remained the highest-rated metrics across all groups and conditions (median 4.00, IQR 4.00-5.00 for GPT-aided vs median 4.00, IQRs ranging from 3.00-4.00 to 4.00-5.00 for human-only), showing minimal change with GPT support and suggesting that these were already a strength among respondents. In contrast, Completeness and Empathy saw notable improvements with GPT, particularly pronounced in CHEWs and RNs, with median Completeness scores increasing from 3.00 (IQR 2.00-4.00) to 4.00 (IQR 3.00-4.00) for CHEWs, and from 3.00 (IQR 3.00-4.00) to 4.00 (IQR 3.00-4.00) for RNs. Median Empathy scores similarly improved from 3.00 (IQR 2.00–4.00) to 4.00 (IQR 2.00–4.00) among CHEWs, and from 3.00 (IQR 2.00–4.00) to 4.00 (IQR 3.00–4.00) among RNs (all *P*<.001). Contextualization increased from median 3.00 (IQR 3.00-4.00) to 4.00 (IQR 3.00-5.00) among CHEWs only, while Overall Preference increased from median 3.00 (IQR 2.00-4.00) to 4.00 (IQR 3.00–4.00) among CHEWs and from 3.00 (IQR 3.00–4.00) to 4.00 (IQR 3.00–4.00) among RNs (all *P*<.001). Although descriptive median scores changed little among MDs, inferential analyses indicated statistically significant improvements across all evaluated domains. These findings suggest GPT notably enhanced response quality across all professional groups, with the greatest descriptive improvements observed among CHEWs and RNs.

**Table 5.** Comparison of clinician-evaluated GPT-aided and human-only responses across 6 quality metrics, stratified by frontline health worker group (CHEWs^a^, MDs^b^, and RNs^c^), reported as median (IQR), from paired responses generated in an observational study in Lagos, Nigeria (2024).^d^

Metric: frontline health worker group		GPT-aided, median (IQR)	Human-only, median (IQR)	*P* value
Accuracy				
CHEWs		4.00 (4.00-5.00)	4.00 (3.00-4.00)	<.001
MDs		4.00 (4.00-5.00)	4.00 (4.00-4.00)	<.001
RNs		4.00 (4.00-5.00)	4.00 (3.00-4.00)	<.001
Empathy				
CHEWs		4.00 (2.00-4.00)	3.00 (2.00-4.00)	<.001
MDs		4.00 (3.00-4.00)	4.00 (3.00-4.00)	.009
RNs		4.00 (3.00-4.00)	3.00 (2.00-4.00)	<.001
Safety				
CHEWs		4.00 (4.00-5.00)	4.00 (3.00-4.00)	<.001
MDs		4.00 (4.00-5.00)	4.00 (4.00-5.00)	<.001
RNs		4.00 (4.00-5.00)	4.00 (3.00-4.00)	<.001
Contextualization				
CHEWs		4.00 (3.00-5.00)	3.00 (3.00-4.00)	<.001
MDs		4.00 (4.00-5.00)	4.00 (3.00-4.00)	<.001
RNs		4.00 (3.00-5.00)	4.00 (3.00-4.00)	<.001
Completeness				
CHEWs		4.00 (3.00-4.00)	3.00 (2.00-4.00)	<.001
MDs		4.00 (3.00-4.00)	4.00 (3.00-4.00)	<.001
RNs		4.00 (3.00-4.00)	3.00 (3.00-4.00)	<.001
Overall preference				
CHEWs		4.00 (3.00-4.00)	3.00 (2.00-4.00)	<.001
MDs		4.00 (4.00-5.00)	4.00 (3.00-4.00)	<.001
RNs		4.00 (3.00-4.00)	3.00 (3.00-4.00)	<.001

a CHEW: community health extension workers.

b MD: medical doctor.

c RN: registered nurse.

d Descriptive statistics are presented as median (IQR). *P* values were obtained from stratified linear mixed-effects models comparing GPT-aided and human-only responses within each frontline health worker group, with frontline health worker respondent included as a random effect and clinical rater included as a fixed-effect covariate.

#### Interrater Reliability of Clinical Evaluations Across Metrics: GPT-Aided vs Human-Only Responses

The consistency of clinical ratings across 6 evaluation metrics was assessed using Fleiss’ Kappa, which quantifies interrater agreement for categorical ratings by 4 raters (Table 6). Kappa values range from 0 to 1, with values between 0.41‐0.60 indicating moderate agreement and 0.61‐0.80 indicating substantial agreement. Among all metrics, overall preference showed the highest interrater reliability, with GPT-aided responses achieving a Fleiss’ Kappa of 0.77 (95% CI 0.73‐0.81), slightly higher than human-only responses at 0.72 (95% CI 0.61‐0.79). Similarly, the Completeness metric demonstrated substantial agreement, with GPT-aided responses at 0.71 (95% CI 0.64‐0.76) and human-only at 0.66 (95% CI 0.53‐0.72). Accuracy, Empathy, and Contextualization all showed moderate consistency, while the Safety metric showed the lowest interrater agreement in both groups.

**Table 6.** Interrater agreement among clinical evaluators for GPT-aided and human-only responses across quality metrics, measured using Fleiss’ kappa with 95% CIs, in an observational study in Lagos, Nigeria (2024).

Metric	GPT-aided, Fleiss κ (95% CI)	Human-only, Fleiss κ (95% CI)
Accuracy	0.60 (0.52‐0.66)	0.62 (0.53‐0.69)
Empathy	0.56 (0.30‐0.70)	0.61 (0.38‐0.73)
Safety	0.50 (0.41‐0.57)	0.44 (0.35‐0.58)
Contextualization	0.58 (0.47‐0.66)	0.55 (0.43‐0.64)
Completeness	0.71 (0.64‐0.76)	0.66 (0.53‐0.72)
Overall Preference	0.77 (0.73‐0.81)	0.72 (0.61‐0.79)

#### Computational Linguistics

The GPT-aided responses were 60% longer than human-only responses, and this difference was statistically significant (mean word count ~120, 95% CI 111.63‐128.29 vs mean ~75, 95% CI 69.90‐79.95; *t* test_1048_=9.09; *P*<.001; difference, 60.1%). Moreover, the GPT-aided responses had higher Flesch grade levels (mean 13.42, SD 4.07 vs mean 11.77, SD 7.41; *t* test_1048_=4.48; *P*<.001; difference, 14.1%), indicating greater linguistic complexity. Their polarity scores were comparable (mean 0.13, SD 0.13 vs mean 0.14, SD 0.18; *t* test_1048_=-1.28; *P*=.20; difference, -8.7%), whereas the GPT-aided responses were more subjective (mean 0.47, SD 0.15 vs mean 0.45, SD 0.20; *t* test_1048_=2.28; *P*=.02; difference, 5.4%).

### Nonclinician Evaluation

Across all nonclinician evaluations (N=1024), GPT-aided responses were descriptively preferred across all themes. The strongest preference was observed for Trust, where GPT-aided responses were favored in 60.0% (614/1024) of assessments. For both Caring and Ease of Understanding, GPT-aided responses were preferred in 57.6% (590/1024) of the assessments.

When stratified by frontline health workers cohorts, this effect was most pronounced for CHEW responses, where 75.0% (192/256) of evaluators found the GPT-aided responses more caring, 71.9% (184/256) easier to understand, and 70.3% (180/256) more trustworthy, representing the highest preference levels observed across all FLW groups. Preferences on GPT-aided responses were lower but still consistent for RN and MD responses. For RN responses, GPT-aided responses were rated highest for Trust (211/384, 54.9%), with similar ratings for Caring and Ease of Understanding (both 199/384, 51.8%). A similar pattern was observed for MD responses, with Trust rated highest (223/384, 58.1%), followed by Ease of Understanding (207/384, 53.9%) and Caring (199/384, 51.8%) (Figure 3).

**Figure 3.**
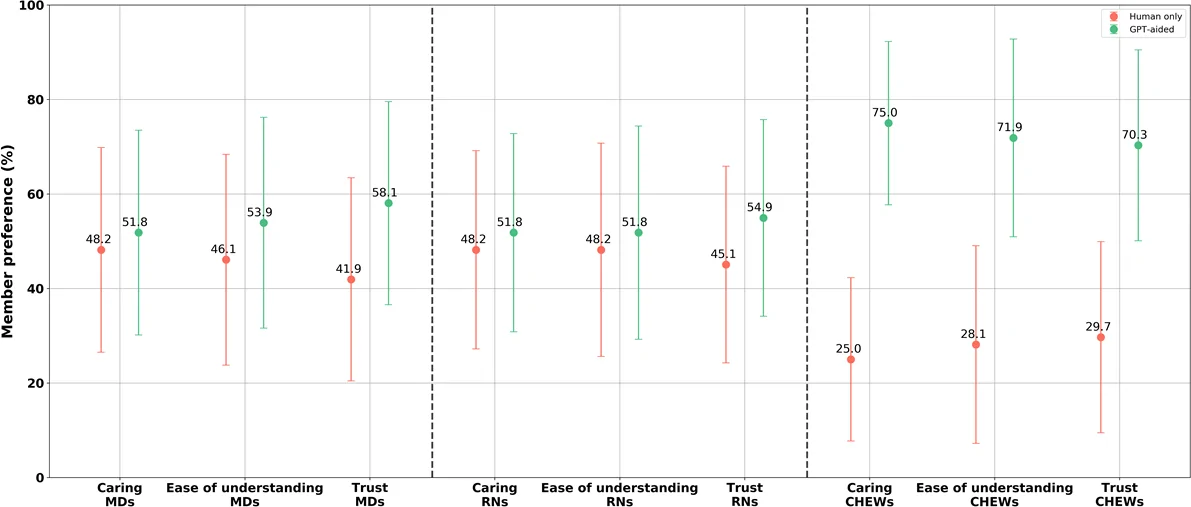
Nonclinician preferences (n=8) for GPT-aided (green) vs human-only (orange) responses across 3 themes (caring, ease of understanding, and trust), stratified by frontline health worker group (MDs, RNs, CHEWs), in an observational study in Lagos, Nigeria (2024). CHEW: community health extension worker; MD: medical doctor; RN: registered nurse.

GEE logistic regression models indicated significantly greater odds of GPT-aided responses being preferred for Trust (OR 1.50, 95% CI 1.04‐2.15; *P*=.03) and Ease of Understanding (OR 1.36, 95% CI 1.05‐1.77; *P*=.02), but not Caring (OR 1.36, 95% CI 0.95‐1.95; *P*=.09).

For CHEW-authored responses, GEE models indicated significantly greater odds of GPT-aided responses being preferred across all 3 evaluation themes: Caring (OR 3.00, 95% CI 1.33‐6.76; *P*=.008), Trust (OR 2.37, 95% CI 1.14‐4.91; *P*=.02), and Ease of Understanding (OR 2.56, 95% CI 1.59‐4.11; *P<*.001). For MD responses, preference was statistically significant only for Trust (OR 1.39, 95% CI 1.09‐1.75; *P*=.007), while no significant preference was observed for Caring (OR 1.08, 95% CI 0.78‐1.48; *P*=.65) and Ease of Understanding (OR 1.17, 95% CI 0.96‐1.43; *P*=.12). For RN responses, no statistically significant preference for GPT-aided responses was observed for Caring (OR 1.08, 95% CI 0.78‐1.49; *P*=.66), Trust (OR 1.22, 95% CI 0.87‐1.72; *P*=.25), or Ease of Understanding (OR 1.08, 95% CI 0.74‐1.56; *P*=.70).

## Discussion

### Principal Findings

The GPT-aided responses consistently outperformed the human-only responses across all evaluated metrics: Accuracy, Empathy, Safety, Contextualization, Completeness, and Overall Preference (Figure 2). These findings align with other studies that have compared ChatGPT’s performance to that of human experts in domains such as oncology-related patient care [27] or ophthalmology [28] and even physician responses on social media [25]. Notably, our data indicate that ChatGPT’s advantages remained consistent regardless of the frontline health workers group level (MDs, RNs, or CHEWs), even though scores were highest for MDs in both human-only and GPT-aided responses (Table 5). These results point to ChatGPT as a potentially powerful supplementary resource in low-resource settings, capable of improving the quality of responses to patients’ health inquiries.

### Comparison to Previous Work

Previous studies have highlighted the potential of AI to improve efficiency and quality of care by automating administrative tasks, providing decision support, and enhancing patient engagement [9,10]. Our results empirically support these claims, showing AI, used as a complementary tool within a human-in-the-loop workflow, may help bridge knowledge and resource gaps in FLWs’ knowledge, resources, or skill capacity common in low- and middle-income countries (LMICs) [29,30]. While MDs maintained the highest scores, ChatGPT notably elevated CHEWs’ and RNs’ responses near MD levels (Table 5). Beyond accuracy, ChatGPT improved empathy and safety scores, demonstrating its ability to adopt a patient-friendly tone despite being designed for broader use [16]. Findings further suggest that LLMs may provide contextually appropriate guidance across language and local settings, supporting frontline workers even in non-English environments [14]. This underscores the potential of LLM-assisted workflows to reduce performance disparities in virtual coaching and general health Q&A in LMICs when integrated as a complementary resource alongside trained frontline workers.

### User Perspectives

Nonclinician evaluators showed a greater overall preference for GPT-aided responses across all three evaluation themes, with the highest preference observed for Trust (Figure 3). Preference remained statistically significant for Trust and Ease of Understanding, whereas the higher preference observed for Caring did not reach statistical significance. These findings suggest that GPT-aided responses may enhance key dimensions of patient-centered communication, particularly perceived trustworthiness and structural clarity. This aligns with findings that people find ChatGPT’s responses more competent and trustworthy [27]. Trust is critical for technology acceptance in health care, and it is an influential factor in users’ acceptance of technology such as ChatGPT for their health care needs [31].

A pivotal finding was the marked divergence in preference observed for responses authored by CHEWs compared to other provider groups. GEE logistic regression demonstrated that the effect of GPT assistance (ChatGPT-3.5) varied by professional designation, with significant preferences across all evaluated domains for responses authored by CHEWs, whereas for MD responses the effect was limited to Trust and no significant preferences were observed for RN responses after accounting for evaluator clustering. This pattern suggests that GPT assistance may provide the greatest communication benefit for frontline health workers with comparatively less formal clinical training, while offering more modest gains for clinicians who may already possess stronger baseline communication skills. Given that CHEWs are often the first or sole point of contact for patients in many LMICs, these findings suggest that AI-assisted communication may enhance the perceived quality and trustworthiness of frontline care. In resource-constrained environments, such tools have the potential to act as a practical equalizer by supporting frontline health workers to deliver communication that more closely reflects higher levels of clinical expertise, thereby helping to bridge gaps in access to specialist care.

Most frontline health workers found ChatGPT user-friendly, efficient, and helpful in improving response speed and quality (Table 1). These findings suggest that integrating LLMs into clinical workflows may improve perceived efficiency and support information retrieval, although this was based on self-reported experience rather than objectively measured time or workload. The findings highlight opportunities to develop specialized LLM health chatbots, fine-tuned with up-to-date clinical guidelines and localized data, to address misinformation and tailor outputs for safety. Such models could further narrow quality gaps among health care workers providing accurate and contextualized advice, especially in resource-constrained LMIC settings.

Moreover, the study found that although GPT-aided responses had higher Flesch-Kincaid grade levels, indicating greater linguistic complexity, nonclinicians still maintained a statistically significant preference for them regarding Ease of Understanding (*P*=.02). This may reflect limitations of traditional readability formulas, which primarily measure sentence length and word complexity but do not capture clarity, structure, or conversational tone. As observed in our results, GPT-aided responses were 60% longer than human-only responses, likely providing more detailed explanations and contextual information, which may have enhanced perceived understanding among nonclinicians. This aligns with findings from recent evaluations in LMICs, where LLM-generated responses were consistently rated higher than clinician responses due to their structured and comprehensive nature, while clinicians’ briefer answers were associated with omission of important information, even with these evaluators being trained clinicians [14].

However, the greater length and linguistic complexity of GPT-generated responses highlight the importance of considering health literacy when implementing LLM-assisted health communication, particularly in LMIC settings. Although readability metrics objectively characterize linguistic complexity, the implications of these findings for patient understanding cannot be determined from this study and warrant further investigation in real-world settings. Practical implementation strategies are needed to prompt models to produce shorter plain-language responses, incorporating health literacy principles into prompt design and validating outputs with lay reviewers. To ensure equitable benefits, future integration must prioritize “plain-language” prompt engineering and “human-in-the-loop” workflows. Fine-tuning models on localized clinical guidelines and indigenous languages will be essential to mitigate misinformation and ensure that AI-driven communication is culturally and medically safe for the Nigerian context.

### Safety and Risks

Despite promising results, caution is warranted. LLMs can generate misinformation (hallucinations) [32,33] and reflect biases in training data. A recent evaluation of LLMs has found that over 20% of the chatbot responses were problematic, including unsafe, factually incorrect, or potentially misleading advice to patient questions [34]. Although clinicians rated GPT-aided responses as safer, rigorous validation and oversight remain essential, especially for high-risk clinical scenarios involving critical diagnoses and treatments. Ongoing efforts to detect and mitigate hallucinations must be integrated into clinical practice to address any inaccuracies [35]. In this study, evaluators were not given specific instructions to identify or automatically penalize hallucinations; instead, factual inaccuracies or potentially misleading information were assessed within the existing evaluation domains, particularly Accuracy and Safety.

In addition, comparatively lower interrater agreement for the Safety metric (Fleiss’ κ=0.44‐0.50) relative to other evaluation metrics for both human-only and GPT-aided responses suggests that assessing the safety of health advice may involve greater subjectivity. Differences in clinicians’ risk tolerance, interpretation of contextual adequacy, and expectations regarding precautionary language or referral guidance may contribute to this variability. These findings highlight the need for clearer evaluation frameworks and standardized criteria when assessing the safety of digital health advice.

### Adoption

Frontline health workers’ skills and readiness to adopt AI are critical. While two-thirds had telemedicine experience and found ChatGPT intuitive, many were new to LLMs, and nearly one-third still trusted traditional resources more. Strengthening their capacity to engage with AI in clinical decision-making will be critical for successful integration into routine care. Targeted AI training and investments in appropriate human-in-the-loop oversight can facilitate safe adoption, balancing risk mitigation with the potential to enhance frontline health care delivery.

### Limitations

The study has some limitations. First, the questions were sourced from those posed by women of reproductive age at a single outreach event in Lagos on a limited number of health topics. The questions may not entirely reflect the full diversity of people’s concerns or the entirety of queries encountered during coaching sessions. However, the number of question-and-answer pairs analyzed in this study was similar to other studies on similar topics [11,30]. Further studies can build on this to analyze a diverse set of responses from a more proportionally representative sample.

Second, the use of ChatGPT-3.5, a model with a 2022 knowledge cutoff, may not fully reflect the capabilities of more advanced LLMs available at the time of the study in 2024. However, ChatGPT-3.5 represented one of the most widely available versions of the tool to the public during the study period.

Third, the number of frontline health workers, though professionally diverse, was relatively small and drawn only from Lagos State. Most participants were also relatively early in their careers, with fewer than 5 years of practice. As a result, the findings may not fully capture the perspectives of more experienced providers or be generalizable to the wider population of FLWs in Nigeria or other low-resource settings.

Fourth, the selection of a nonclinician and a clinical evaluation panel. The panel included clinicians with advanced training across multiple medical specialties to ensure that the clinical appropriateness and depth of responses could be assessed across different health topic domains. The panel did not include CHEWs, despite them being one of the frontline health workers included in the study. Hence, response quality was evaluated primarily from the perspective of doctors and nurses, which may not fully reflect the scope of practice of CHEWs. Future studies could include CHEW evaluators to capture perspectives across frontline health worker roles. The nonclinician evaluation panel had a relatively high level of educational attainment, with 75% (6/8) holding Bachelor’s degrees. Although tertiary education was not an inclusion criterion, this may reflect the requirement for English fluency and minimum secondary education, as well as recruitment from a digitally engaged user base. As a result, the findings related to ease of understanding and trust may not be fully generalizable to populations with lower literacy levels. Additionally, because nonclinician evaluators were recruited from the organization’s active user base through their health coaches, some degree of social desirability bias in subjective evaluation domains, such as Trust, cannot be completely excluded. Related to the nonclinician sampling design, the nonclinician preference evaluation sampled responses in a 6:6:4 ratio (MDs:RNs:CHEWs) per question, which does not reflect the equal 12:12:12 enrollment distribution, resulting in CHEW-authored responses contributing fewer assessments (256) compared with MD- and RN-authored responses (384 each). Given that CHEWs demonstrated the strongest preference for GPT-aided responses across all themes, this underrepresentation likely results in a conservative estimate of the overall GPT preference signal.

Fifth, variability in prompting techniques among participants may have influenced the results. Participants were not provided with a standardized prompting protocol when interacting with ChatGPT, resulting in differences in how questions were entered. This approach measures real-world usage rather than controlled performance as our objective was to observe how FLWs would naturally interact with an LLM in a high-pressure, real-world environment without specialized training. While most participants entered questions verbatim, some rephrased queries based on their interpretation. Additionally, differences in participants’ prior experience with AI tools may have contributed to this variability, as those with greater AI literacy may have been more adept at formulating effective prompts, further conflating model performance with individual user capability. This variability in prompt formulation and capacity of the participants may have affected response quality, such that outcomes may reflect both the model’s capabilities and participants’ individual prompting approaches. We acknowledge that this introduces “prompt engineering skill” as a mediating variable. However, we believe this provides a more pragmatic assessment of the tool’s utility in resource-constrained settings where standardized training may not be immediately feasible.

Sixth, the a priori power calculation assumed independence across all 1080 responses, not accounting for the intraclass correlation (ICC) arising from 30 responses clustered within each of the 36 participants. As the ICC was unknown prior to data collection, a cluster-adjusted sample size could not be prospectively determined, and the 1080 response target should be interpreted as a minimum feasibility estimate. The use of linear mixed-effects models and GEE in the final analyses accounted for the clustered data structure during statistical inference but does not retrospectively address the assumptions underlying the original sample size calculation. In addition, while the GEE models specified evaluator ID as the primary clustering variable, residual dependence arising from the nesting of response pairs within individual frontline health workers and within specific questions was not explicitly modeled.

Finally, the study design may have introduced additional sources of bias related to workload, question order, and evaluation conditions. Participants and evaluators described their respective tasks as cumbersome. Bias may have been introduced by design-type, “learning effect” or “fatigue effect.” Participants completed the human-only responses before generating GPT-aided responses, which may have introduced a learning or anchoring effect as participants became more familiar with the questions during the first round. In the context of this study’s fixed-sequence design, the model functioned as a complementary resource rather than a standalone primary resource. While clinicians did not use the model to edit their initial human drafts, their prior engagement with the prompts may have influenced their subsequent prompting strategies. This reflects a realistic clinical workflow where AI supplements professional knowledge, even if it doesn’t show us how the AI would perform completely on its own.

In addition, for evaluators, although responses were randomized, anonymized, and blinded by type to avert bias, higher differences in response length (60%) and higher linguistic complexity of GPT-aided responses (13.42 vs 11.77) may have allowed some evaluators to infer the response origin, potentially introducing observer bias. Although readability analyses objectively quantified the linguistic complexity of the generated responses, this study did not assess comprehension or readability among the intended end users. Consequently, the practical implications of the observed readability levels for patient understanding cannot be directly inferred from this study. Furthermore, the volume of responses evaluated (total 1080 responses) over a relatively short 5-day period may have contributed to cognitive fatigue among clinical evaluators, which could have influenced scoring consistency and reliability. Evaluations were conducted asynchronously, allowing evaluators to complete assessments at their own pace rather than within fixed sessions, which may have reduced acute fatigue effects. Importantly, interrater reliability remained moderate to substantial across all evaluation metrics for both human-only and GPT-aided responses (Fleiss’ kappa range: 0.44‐0.77), suggesting that scoring consistency was maintained despite the intensive evaluation period (Table 6).

### Recommendations

First, evidence from our study showed that 41.7% of frontline health workers had no prior experience with LLMs and relied on traditional resources (Table 1). Targeted training is needed to build confidence and practical skills in using LLM tools in the daily clinical workflow. Second, implementing human-in-the-loop approaches can ensure safe adoption, allowing clinicians to validate AI outputs and scale LLM integration effectively. Third, future studies could conduct larger and more diverse and representative samples of FLWs to improve the generalizability of the findings, and finally, future research should also evaluate the performance of different LLMs, beyond ChatGPT, as differences in model architecture may influence response quality, safety, and effectiveness.

### Conclusions

GPT-aided responses were rated superior by clinicians and preferred by nonclinicians within the study sample, which consisted of a relatively highly educated and digitally engaged population, highlighting opportunities to expand AI-assisted healthcare communication. These findings suggest that ChatGPT-3.5 may serve as a valuable complementary tool for frontline health workers in low-resource settings by improving the quality, consistency, and perceived trustworthiness of patient-facing communication. However, important considerations remain. GPT-generated responses were longer and more linguistically complex, which may limit accessibility for lower-literacy populations. As voice-enabled interfaces for such models become more widely available, these tools may help bridge literacy and usability barriers by allowing both patients and frontline health workers to interact with systems through spoken language. This has the potential to improve accessibility, reduce cognitive burden, and support more inclusive use in resource-constrained settings. In addition, LLM outputs can be susceptible to inaccuracies or misleading information, necessitating continued human oversight and validation. To address these challenges, safe and effective integration will require careful prompt design, adaptation to local contexts, and human-in-the-loop approaches. Further research is needed to evaluate performance, safety, and usability across more diverse, representative, and resource-constrained populations, including lower-literacy settings.

## Supplementary material

10.2196/86578 Multimedia Appendix 1 Health questions.

10.2196/86578 Multimedia Appendix 2 Evaluation rubric for clinical evaluators.

## Abbreviations

CHEW: community health extension worker
GEE: generalized estimating equation
ICC: intraclass correlation
LLM: large language model
LMIC: low- and middle-income country
LMM: linear mixed-effects model
MD: medical doctor
ND: National Diploma
RN: registered nurse
WHO: World Health Organization
