# Supplementary material for: Evaluating Frontline Health Workers’ Responses to Patient Inquiries With and Without Large Language Model Support in Nigeria: Observational Study
Source: J Med Internet Res. 2026 Nov 5;28:e86578. doi: 10.2196/86578 (PMC13739204; doi:10.2196/86578)
Supplement: Supplementary file 1 [file jmir-v28-e86578-s001.pdf]

Supplementary Table 1: Health questions

Topic	Question Number	Question
Maternal Health	1	I have been trying to get pregnant after doing family planning. I did scan, they said no fibroid. Why can't I take in?
Maternal Health	2	I have been having irregular period. Sometimes my period comes for 6 days, sometimes 3 days, and sometimes 4 days. Then when it comes for 6 days, I see it twice a month and the other times once a month.
Maternal Health	3	I have heard about cryptic pregnancy. Is it in the gene, I mean can it happen to everyone? For instance, giving birth to twin is in the gene. Is this the same for cryptic pregnancy?
Neonatal Health	4	What can I use for my baby for him to grow up strong and for his skin to be clear?
Neonatal Health	5	My baby's temperature is usually high at night but comes down in the morning. Should I be worried?
Neonatal Health	6	My baby breathes as though something is blocking her nostrils and this only happens in the night. When she sleeps in the afternoon, it is not the same. I don't just like how she breathes as it gets me worried. What can I do?
Sexually Transmitted Infections	7	Lack of regular sexual intercourse does it affect my health in any way?
Sexually Transmitted Infections	8	If you use a public toilet and your body quickly reacts to get an infection, how do you treat it?
Sexually Transmitted Infections	9	I heard condoms have holes in them, do they protect against infection?
Family Planning	10	I don't want to bear a child again as I have 3, what family planning can I do?
Family Planning	11	I plan to have family planning after my next child, but I don't like it, what do I do?
Family Planning	12	I have been on family planning since 2021 but sometimes I don't use to see my period. What should I do?
Neonatal Health	13	Someone said if I don't circumcise my baby boy that he would not have a normal life. Is this true?
Sexually Transmitted Infections	14	I have been having recurrent whitish discharge for over three years, what can I do?
Family Planning	15	My family planning has been for 3 years initially I wasn't seeing my period in the first year, now I see my period every other month, what does this mean as I don't want to get pregnant?
