# Supplementary material for: Evaluating Frontline Health Workers’ Responses to Patient Inquiries With and Without Large Language Model Support in Nigeria: Observational Study
Source: J Med Internet Res. 2026 Nov 5;28:e86578. doi: 10.2196/86578 (PMC13739204; doi:10.2196/86578)
Supplement: Supplementary file 2 [file jmir-v28-e86578-s002.pdf]

Evaluation Metric	Description	Scoring Scale
Accuracy	Is the response provided accurate?	1 = Completely inaccurate<br>2 = Mostly inaccurate<br>3 = Approximately equal accurate & inaccurate<br>4 = Mostly accurate<br>5 = Completely accurate
Empathy	Does the medical professional show understanding of and attempt to put themself in the woman's position?	1 = No understanding/empathetic<br>2 = Slightly understanding/empathetic<br>3 = Moderately understanding/empathetic<br>4 = Understanding/empathetic<br>5 = Very understanding/empathetic<br>N/A = Not applicable
Safety	The response would not cause any form of harm.	1 = Strongly disagree<br>2 = Disagree<br>3 = Neutral<br>4 = Agree<br>5 = Strongly agree<br>N/A = Not applicable
Contextualization	Does the response fit the local Nigerian context?	1 = Completely irrelevant<br>2 = Mostly irrelevant<br>3 = Moderately irrelevant<br>4 = Mostly relevant<br>5 = Completely relevant<br>N/A = Not applicable
Completeness	Does the response address all aspects of the question?	1 = Not complete at all<br>2 = Mostly incomplete<br>3 = Moderately complete<br>4 = Mostly complete<br>5 = Complete<br>N/A = Not applicable
Overall Preference	Overall, how do you rate the quality of the response?	1 = Very poor<br>2 = Poor<br>3 = Acceptable<br>4 = Good<br>5 = Very good
